# Supplementary material for: Robust deep learning object recognition models rely on low frequency information in natural images
Source: PLoS Comput Biol. 2023 Mar 27;19(3):e1010932. doi: 10.1371/journal.pcbi.1010932 (PMC10079058; doi:10.1371/journal.pcbi.1010932)
Supplement: S1 Appendix — (PDF) [file pcbi.1010932.s001.pdf]

Supporting information

S1 Appendix. **Additional analysis and results.**

Appendix

Common corruption categorization

Fourier analysis is performed for the perturbations induced by common corruptions in the CIFAR10-C dataset (at severity 5). All 15 corruptions are divided loosely into three categories based on their dominant frequencies (Tab. A)

category	corruptions
low	snow, frost, fog, brightness, contrast
medium	motion_blur, zoom_blur, defocus_blur, glass_blur, elastic_transform, jpeg_compression, pixelate
high	gaussian_noise, shot_noise, impulse_noise

Table A. Categorization of common corruptions. 15 types of corruptions[1] are divided into 3 categories based on the average frequency estimated from the Fourier spectrum of the perturbations (Fig. A).

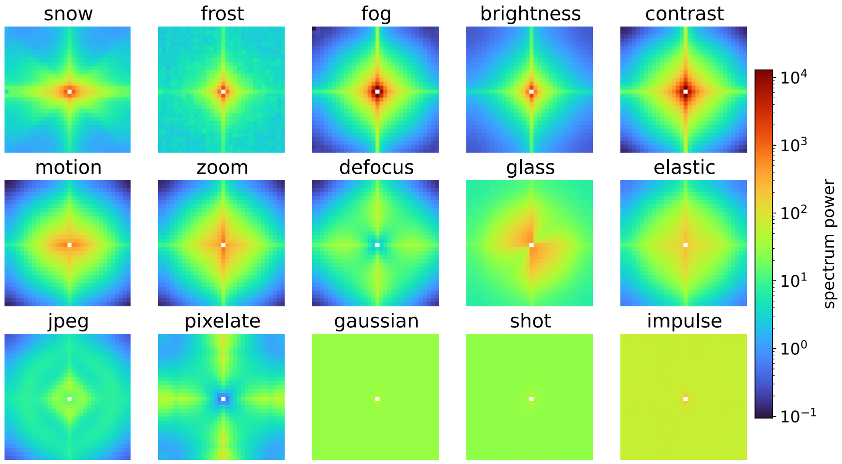

Figure A. Corruption spectrum for the CIFAR10-C dataset. Fourier power spectra are plotted for all different common corruptions. Color maps are shared across panels.

Hybrid image experiment for monkey regularized model

Frequency bias is compared between a monkey-response-regularized VGG model and a baseline model through the experiment using hybrid images created from Tiny-ImageNet dataset. Though a weaker effect compared with the mouse-regularization result, we found the reversing frequency for ‘neural’ model is smaller than that of ‘base’ model, suggesting a low frequency bias induced by neural regularization.

Details of robust models

Details of models trained for CIFAR10, including six baseline models, seven models trained for adversarial robustness, two models trained for common corruption robustness and two models using preprocessing are shown in Tab. B. The minimal perturbation size needed to change prediction are listed for each model, with mean and standard deviation computed from 1000 images.

Details of models trained for ImageNet, including one baseline model, two models trained for adversarial robustness and six models trained for common corruption robustness are shown in Tab. C.

Analysis on neural similarity matrix

Previous work[23] demonstrated that models regularized with neural similarity matrix are more robust against multiple types of pixel noise as well as adversarial attacks. To understand why this type of neural regularization works, we analyzed the neural

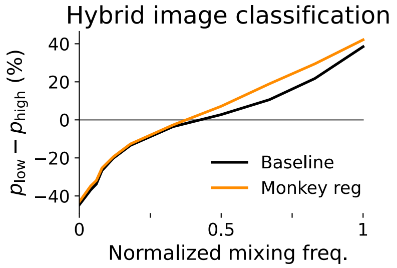

Figure B. Probing frequency sensitivity of the monkey regularized model using hybrid images. Results are presented similar to Fig. 3b in main text.

Type	Model name	Architecture	Test accuracy on CIFAR10	ε (L_∞)
baseline	Base-WideRes[2]	WideResNet-28-10	94.78%	1.17 (0.02)
	Base-Res[3,4]	ResNet-56	94.37%	1.03 (0.02)
	Base-VGG[4,5]	VGG-19	93.91%	1.75 (0.02)
	Base-MobNet[4,6]	MobileNetV2-x1-4	94.22%	1.03 (0.02)
	Base-ShufNet[4,7]	ShuffleNetV2-x2-0	93.81%	1.52 (0.02)
	Base-RepVGG[4,8]	RepVGG-a2	94.98%	1.50 (0.02)
adversarial	Rebuff21[9]	WideResNet-70-16	92.23%	16.88 (0.21)
	Gowal20[10]	WideResNet-70-16	91.10%	17.86 (0.23)
	Wu20[11]	WideResNet-28-10	88.25%	18.45 (0.26)
	Zhang20[12]	WideResNet-28-10	89.36%	16.89 (0.23)
	Carmon19[13]	WideResNet-28-10	89.69%	16.80 (0.23)
	Sehwag20[14]	WideResNet-28-10	88.98%	16.70 (0.24)
	Cui20[15]	WideResNet-34-20	88.70%	15.33 (0.22)
corruption	Hendrycks20[16]	ResNeXt29-32x4d	95.83%	1.52 (0.02)
	Kireev21[17]	PreActResNet-18	94.77%	3.53 (0.05)
preprocess	Blur ($\sigma = 1.5$)	ResNet-18	90.66%	2.30 (0.04)
	PCA ($K = 512$)	ResNet-18	89.85%	2.69 (0.04)

Table B. Models trained for CIFAR10. Six baseline models, seven models trained for adversarial robustness, two models trained for common corruption robustness, and two models with simple preprocessing are compared in this study.

similarity matrix that characterizes the geometry of mouse V1 representation. We obtain neural responses of natural images through a well trained predictive model[24], and denote the population response to image i as r_i. The dimension of vector r_i is the number of neurons. Neural similarity matrix S^{neural} is defined as the cosine similarity of mean-corrected responses $r_1, \dots, r_N$ for N images,

$$S_{ij}^{\text{neural}} = \frac{\tilde{r}_i \cdot \tilde{r}_j}{\|\tilde{r}_i\| \|\tilde{r}_j\|}, \tag{1}$$

in which $\tilde{r}_i = r_i - \bar{r}$ is the population response to image i subtracted by mean response.

A first thing to notice is that the neural similarity matrix is low rank. For example, the one shown in Fig. C is a 5000×5000 matrix from 5000 images, but a rank-204 approximation can explain more than 90% of its variance. To account for 99% of the variance, a matrix of rank 1452 is sufficient. The result is not due to a small number of neurons. In fact, the neural response vector r_i used in this example is a union over 8 different scans, containing more than 40,000 recorded units. The low rank nature of S^{neural} shows that the vision system is encoding a small number of features through a highly correlated neuron population.

The next question is, how do these neural features look? Performing eigenvalue decomposition on S^{neural}, we can calculate its eigenvalues $\lambda_1, \lambda_2, \dots, \lambda_N$ ($\lambda_1 > \lambda_2 > \dots > \lambda_N$) and the corresponding eigenvectors $v_1, v_2, \dots, v_N$ ($\|v_i\| = 1$). The i-th neural feature is defined as $f_i = \sqrt{\lambda_i} v_i$. The rank-204 approximation in Fig. C is generated using the first 204 neural features, *i.e.* $\hat{S} = \sum_{i=1}^{204} f_i f_i^{\mathrm{T}}$.

Each neural feature f_i is a vector of the same length as the number of images, and can be treated as a scalar function of images. The first order approximation of f_i is a linear model with respect to the pixel values as input. The linear weight can be

Type	Model name	Architecture	Top-1 test accuracy on ImageNet	ε (L_∞)
baseline	Baseline[18]	ResNet-50	76.13%	0.45 (0.01)
adversarial	L_∞ ($\varepsilon = 4/255$)[18]	ResNet-50	62.42%	10.82 (0.11)
	L_2 ($\varepsilon = 3$)[18]	ResNet-50	57.90%	10.52 (0.10)
corruption	ANT[19]	ResNet-50	76.07%	0.66 (0.01)
	SIN[20]	ResNet-50	74.59%	0.52 (0.01)
	AugMix[16]	ResNet-50	77.54%	0.58 (0.01)
	DeepAugment[21]	ResNet-50	74.59%	0.72 (0.01)
	DeepAug+AugMix[21]	ResNet-50	75.82%	0.90 (0.01)
	Assemble[22]	Assemble-ResNet-50	80.81%	0.23 (0.00)
preprocess	Blur ($\sigma = 3$)	ResNet-50	71.87%	2.11 (0.03)

Table C. Models trained for ImageNet. One baseline model, two models trained for adversarial robustness, six models trained for common corruption robustness and one model trained with blurring preprocessing are compared.

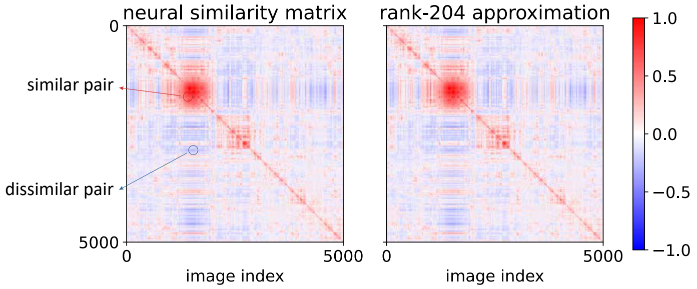

Figure C. The neural similarity matrix and its low rank approximation. Neural responses of 5000 grayscale images are provided by a well trained brain model[24]. The cosine similarities between all pairs of responses are then calculated after subtracting the mean responses. An eigenvalue decomposition of the matrix shows that the first 204 principal components account for more than 90% of the variance. Therefore, a low rank approximation can be constructed based on these components.

easily calculated by solving the regression problem,

$$w_i = \underset{w_i}{\operatorname{argmin}} \left(\left\| f_i - w_i^\mathsf{T} X \right\|^2 + \alpha \|w_i\|^2 \right). \tag{2}$$

Each column of X is a flattened image, and the dimension of w_i vector is the number of pixels. $\alpha \|w_i\|^2$ is a regularization term. The first 16 linear weights w_i are visualized as as spatial maps in Fig. D.

We further analyzed two properties of the linear approximation of neural features. Treating w_is as spatial maps, we can calculate its dominant spatial frequency via Fourier analysis. The results show that the dominant Fourier component of w_i associated with strong neural features are relatively low frequency (Fig. E). Though w_i show certain spatial structure (Fig. D), neural features f_i are nonlinear in general. We quantified the linearity of f_i by how good the linear approximation is, and found that correlation coefficient between f_i and the best linear prediction is high only for the neural features with high eigenvalues (Fig. E).

Variance of model instances with the same architecture

While we majorly investigated the frequency preference of different individual models, one might wonder how big is the variance among models of the same architecture. We therefore computed the half power frequency $f_{0.5}$ (Fig. 4 in main text) and the reverse frequency f_{rev} (Fig. 5 in main text) for a set of models of the same architecture but trained with different random seeds. For simplicity, we only trained 5 'blur' models with ResNet18 backbone on CIFAR10, using the blurring parameter $\sigma = 1.5$. Mean value and standard deviation over 5 random seeds of the half power frequency is $f_{0.5} = 0.181 \pm 0.0047$, and the reverse frequency is $f_{\text{rev}} = 0.241 \pm 0.0057$ (Fig. F). In addition, both metrics correlate strongly with each other, suggesting either one is suitable for characterizing model frequency preference.

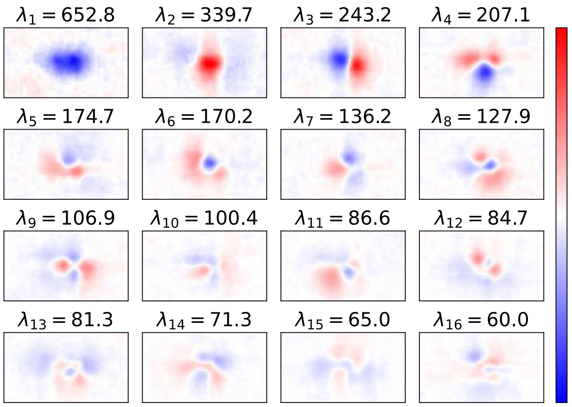

Figure D. Linear approximation of neural features. Neural features are the eigenvectors v_i of the neural similarity matrix properly scaled by corresponding eigenvalues λ_i. Each neural feature is approximated by a linear function on image pixel values, and the linear weight w_i is displayed as a spatial map.

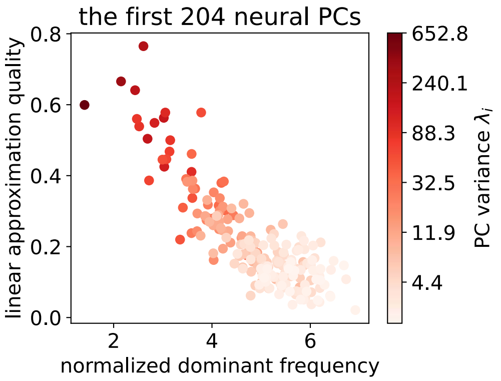

Figure E. Overview of neural features. Properties of the first 204 neural features are visualized with colors indicating the eigenvalue corresponding to each. Each neural feature is approximated by a linear model. The ordinate is the correlation coefficient of linear approximation and the neural features on a hold-out set of images, characterizing how linear the feature is. The abscissa is the dominant frequency of the linear weights when viewed as spatial maps (Fig. D). The results show that neural features, with high eigenvalues, are more linear, and contains lower spatial frequencies.

Model prediction on hybrid images

We reported the probability difference $p_{\text{low}} - p_{\text{high}}$ in Fig. 3 in the main text, here we also show p_{low} and p_{high} respectively for the baseline model on grayscale CIFAR10. When the mixing frequency is in medium range, neither p_{low} or p_{high} is big, and the model classify the image as neither the low-frequency category nor high-frequency one with approximately 50% probability. We also looked at the model confidence, *i.e.* the probability of reported category p_{pred}, and found it is lowest at ambiguous mixing frequency region.

Breakdown robustness to different common corruptions

For simplicity we use the model performance averaged over all common corruption types at all severity levels as the robustness against common corruptions in the main text. However, we already observe that model robustness depends on the Fourier spectrum of different corruptions and we should expect to see some different behavior since models' spatial frequency preference are different. Here we report the breakdown version of the relationship between robustness and frequency preference for CIFAR10-C dataset, *i.e.* Fig. 5 for individual severity level and corruption type. All five severity levels (Fig. H) and five randomly picked corruption types (Fig. I) are shown.

When comparing the results conditioned on different corruption types, we found though the exact pattern of robustness against frequency preference are different, overall the consistency between public models and the 'blur' models holds for all conditions, suggesting that a simple view of filtering out Fourier components explains the robustness in various models for CIFAR10 dataset.

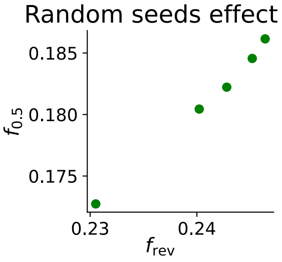

Figure F. Variance of frequency preference index over different random seeds of the same model architecture. Five ResNet18 models (green dots) with blurring preprocessing are trained on CIFAR10, the half power frequency $f_{0.5}$ and reverse frequency f_{rev} are computed for each of them. All values are close to the one reported in Fig. 4c and Fig. 5b in the main text, showing the variance caused by random seeds are smaller than that between different architecture.

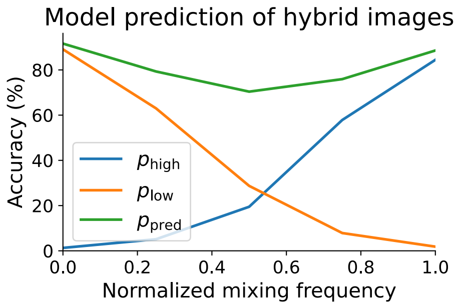

Figure G. Baseline model (in Fig. 3) predictions on hybrid images. Both p_{low} and p_{high} are reported for different mixing frequencies, as well as the model confidence p_{pred}.

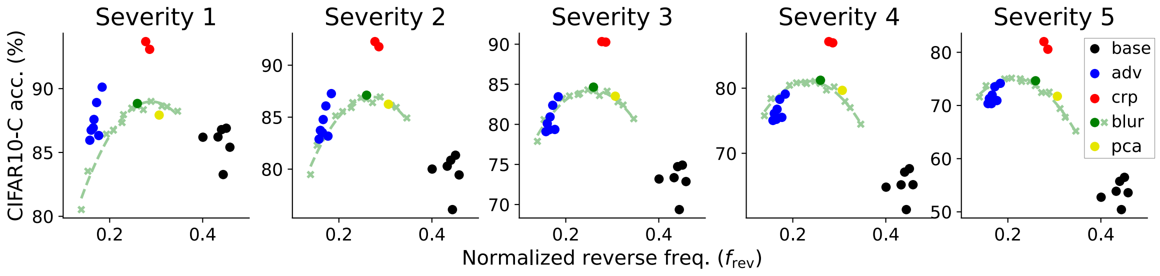

Figure H. Performance on CIFAR10-C against frequency preferences of models at different severity levels. Classification accuracy is marginalized over different corruption types conditioned on each severity level.

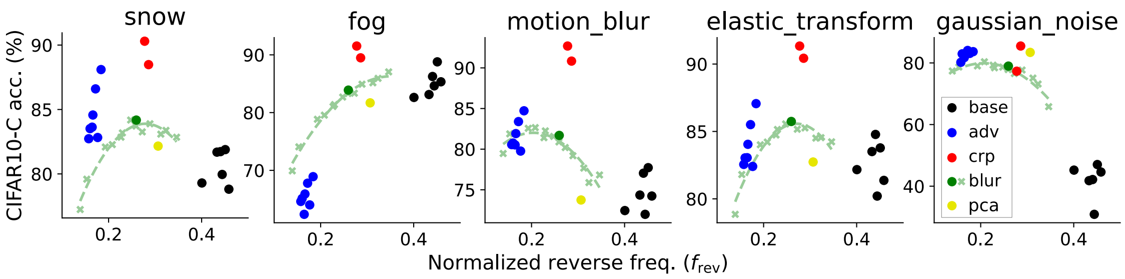

Figure I. Performance on CIFAR10-C against frequency preferences of models against different corruption type. Classification accuracy is marginalized over all severity levels conditioned on each corruption type.

References

1. Hendrycks, D. & Dietterich, T. Benchmarking neural network robustness to common corruptions and perturbations. In *International Conference on Learning Representations* (2019).
2. Croce, F. *et al.* Robustbench: a standardized adversarial robustness benchmark. *arXiv e-prints* (2020).
3. He, K., Zhang, X., Ren, S. & Sun, J. Deep residual learning for image recognition. In *Proceedings of the IEEE conference on computer vision and pattern recognition*, 770–778 (2016).
4. Chen, Y. Pytorch cifar models.
5. Simonyan, K. & Zisserman, A. Very deep convolutional networks for large-scale image recognition. In Bengio, Y. & LeCun, Y. (eds.) *3rd International Conference on Learning Representations, ICLR 2015, San Diego, CA, USA, May 7-9, 2015, Conference Track Proceedings* (2015).
6. Sandler, M., Howard, A., Zhu, M., Zhmoginov, A. & Chen, L.-C. Mobilenetv2: Inverted residuals and linear bottlenecks. In *Proceedings of the IEEE Conference on Computer Vision and Pattern Recognition (CVPR)* (2018).
7. Ma, N., Zhang, X., Zheng, H.-T. & Sun, J. Shufflenet v2: Practical guidelines for efficient cnn architecture design. In *Proceedings of the European Conference on Computer Vision (ECCV)* (2018).
8. Ding, X. *et al.* Repvgg: Making vgg-style convnets great again. In *Proceedings of the IEEE/CVF Conference on Computer Vision and Pattern Recognition (CVPR)*, 13733–13742 (2021).
9. Rebuffi, S.-A. *et al.* Fixing Data Augmentation to Improve Adversarial Robustness. *arXiv e-prints* arXiv:2103.01946 (2021).
10. Gowal, S., Qin, C., Uesato, J., Mann, T. & Kohli, P. Uncovering the Limits of Adversarial Training against Norm-Bounded Adversarial Examples. *arXiv e-prints* (2020).
11. Wu, D., Xia, S.-t. & Wang, Y. Adversarial Weight Perturbation Helps Robust Generalization. *arXiv e-prints* arXiv:2004.05884 (2020).
12. Zhang, J. *et al.* Geometry-aware Instance-reweighted Adversarial Training. *arXiv e-prints* arXiv:2010.01736 (2020). 2010.01736.
13. Carmon, Y., Raghunathan, A., Schmidt, L., Liang, P. & Duchi, J. C. Unlabeled Data Improves Adversarial Robustness. *arXiv e-prints* (2019).
14. Sehwag, V., Wang, S., Mittal, P. & Jana, S. HYDRA: Pruning Adversarially Robust Neural Networks. *arXiv e-prints* (2020).
15. Cui, J., Liu, S., Wang, L. & Jia, J. Learnable Boundary Guided Adversarial Training. *arXiv e-prints* (2020).
16. Hendrycks, D. *et al.* Augmix: A simple method to improve robustness and uncertainty under data shift. In *International Conference on Learning Representations* (2020).
17. Kireev, K., Andriushchenko, M. & Flammarion, N. On the effectiveness of adversarial training against common corruptions. *arXiv e-prints* (2021).
18. Engstrom, L., Ilyas, A., Salman, H., Santurkar, S. & Tsipras, D. Robustness (python library) (2019).
19. Rusak, E. *et al.* A simple way to make neural networks robust against diverse image corruptions. In Vedaldi, A., Bischof, H., Brox, T. & Frahm, J.-M. (eds.) *Computer Vision – ECCV 2020*, 53–69 (Springer International Publishing, Cham, 2020).
20. Geirhos, R. *et al.* Imagenet-trained CNNs are biased towards texture; increasing shape bias improves accuracy and robustness. In *International Conference on Learning Representations* (2019).
21. Hendrycks, D. *et al.* The Many Faces of Robustness: A Critical Analysis of Out-of-Distribution Generalization. *arXiv e-prints* (2020).
22. Lee, J. *et al.* Compounding the Performance Improvements of Assembled Techniques in a Convolutional Neural Network. *arXiv e-prints* arXiv:2001.06268 (2020).
23. Li, Z. *et al.* Learning from brains how to regularize machines. In Wallach, H. *et al.* (eds.) *Advances in Neural Information Processing Systems 32*, 9525–9535 (Curran Associates, Inc., 2019).
24. Sinz, F. *et al.* Stimulus domain transfer in recurrent models for large scale cortical population prediction on video. In Bengio, S. *et al.* (eds.) *Advances in Neural Information Processing Systems 31*, 7199–7210 (Curran Associates, Inc., 2018).
