# Supplementary material for: Robust deep learning object recognition models rely on low frequency information in natural images
Source: PLoS Comput Biol. 2023 Mar 27;19(3):e1010932. doi: 10.1371/journal.pcbi.1010932 (PMC10079058; doi:10.1371/journal.pcbi.1010932)
Supplement: Attachment — Submitted filename: Review_for_PLOS_Comp__Bio.pdf [file pcbi.1010932.s002.pdf]

Review Summary: This paper shows that the deep neural network models that are robust to adversarial images or data augmentations share one common feature of preferring the low frequency information in the natural images. The authors started their analysis from the neural regularized models and found that the mouse-regularized model has a strong preference of the low frequency feature. They then validated that the other publicly available robust models also share this preference and further proposed the blurring preprocessing as a defense strategy against the attacks.

At a high level, I think the main point of this article (robust networks prefer low-frequency features) is indeed supported by the presented evidence. This result will be useful for the community to better understand these robust networks and then propose the networks that are even more robust. However, I think there are several (possibly critical) issues that need to be addressed to make the whole story coherent.

Major issues:

1. The first part of the conclusion in this paper is "the increased model robustness (of the neural regularized models) is partly due to the low spatial frequency preference inherited from the neural representation". However, *this conclusion does not seem general at all.* In fact, the authors only analyzed two neural-regularized networks that are very different from each other in almost all aspects: one is the mouse regularized ResNet-18 trained on CIFAR10, the other is the monkey regularized VGG19 trained on TinyImageNet. Even though only two networks were analyzed, the monkey regularized one barely supports the claim as its preference to low frequency is **very weak** (see Fig. 2.h and 8.b). Given that the two networks are different in their architectures, training datasets, and the animal model used for regularization, it's hard for me to tell why the mouse regularized one supports the claim but the monkey one does not. To address this issue, the authors need to clearly show which factor is the key factor in making these two models so different in whether they prefer the low frequency features through controlling other factors but varying one.

2. This paper also claims that the blurring preprocessing is a good defense strategy for the adversarial attacks and also the augmentations. But the authors only provided the evidence for this on the weaker dataset (CIFAR10) and one specific architecture (ResNet-18). Have the authors tried this method with other architectures (like ResNet-50)? More importantly, what about ImageNet? It seems quite easy to try that on the ResNet-50s the authors have on ImageNet, so it makes me wonder whether the authors have tried and in fact got the opposite or negative results. If indeed so, I think it needs to be mentioned and possibly analyzed to give a better picture about the strength of this preprocessing strategy. In addition, have the authors tried training the networks from scratch but with the blurring in the data augmentation pipeline (but not necessarily as the preprocessing)? This training with blurring idea seems natural to me and

I just wonder whether it will work even better than the post-preprocessing method.

Minor issues:

1. This might be related to the first major issue, but I wonder how the authors address the inconsistent interpretations of the results shown in Fig 2.g and 2.h. In Fig 2.g, the monkey regularized network has a larger performance boost for the high frequency corruptions over the baseline network compared to other corruptions, but Fig 2.h as well as Fig 8.b seems to suggest that the frequency feature of this network is very similar to the baseline network. Does this mean that the $f_{0.5}$ or the f_{rev} metric does not reflect the whole preference to the low frequency features or that the results in Fig 2.g are not about this preference?

2. For some of the results, the authors provided the variance with respect to the test images. How about the variance due to different random seeds during the network training and the weight initialization? If the authors can at least provide some measure about this for the CIFAR10 networks, that would be useful to tell how robust or reliable the effects measured in this paper are.

3. The Fig. 4b and 5a are somewhat confusing to me. The caption mentioned dotted lines, but I cannot find them in the plots.

4. At the end of the first paragraph of page 3, the "Fig. 2c, f" should be "Fig. 2c, g".

Additional Questions: I hope the authors can add the answers of these questions to the discuss part.

1. Can the authors discuss whether certain features of the mouse visual system can explain the result that the mouse regularized network shows the low frequency feature preference?

2. If the low frequency feature preference cannot fully explain the reason why the mouse regularized network is more robust than the baseline network, can the authors discuss the other possible reasons?

3. What is the difference between the results shown in this work compared to the results of the very recent publication also in PLOS Comp. Bio.: "Increasing neural network robustness improves match to macaque V1 eigenspectrum, spatial frequency preference and predictivity"? This recent work claims "robust models had preferred spatial frequency distributions more aligned with the measured spatial frequency distribution of macaque V1 cells", which seems related to the claims by the authors.
