# Supplementary material for: Robust deep learning object recognition models rely on low frequency information in natural images
Source: PLoS Comput Biol. 2023 Mar 27;19(3):e1010932. doi: 10.1371/journal.pcbi.1010932 (PMC10079058; doi:10.1371/journal.pcbi.1010932)
Supplement: Attachment — Submitted filename: ploscb review.pdf [file pcbi.1010932.s003.pdf]

In this work, the authors tackle the issue of adversarial robustness of deep neural network models, and its connection to neuroscience. Recent years have seen enormous progress in the construction of deep learning models for many tasks, including vision, but despite high levels of performance, these models remain highly sensitive to either natural corruption (e.g. noise, occlusion, etc.) or adversarially-generated perturbations (imperceptible noise that can be crafted in such a way that it makes the model fail). Since the brittleness of these models seems at odds with the apparent robustness of biological vision, many have sought inspiration or constraints from natural brains to address these issues, and a number of approaches designed to make models more "brain-like" also appear to make models more robust. In this paper, the authors argue that a large component of this improved robustness comes from biasing models towards lower spatial frequency information.

The paper does a good job of analyzing the robustness of a range of models across a range of different kinds of natural corruptions and adversarial attacks, and showing that there are signs that more robust models (made that way either through neural regularization or other robust training methods) seem to achieve their robustness by preferring low frequency information. The paper uses a hybrid image paradigm to probe the relative high-vs.-low frequency, which provides some insight into the frequency preferences of various models. I think that these kind of "neuroscience-style" analyses are a welcome addition to the deep learning literature, and provide good insights (and a framework for interrogating future models as well).

I think that this is a useful contribution to the literature, though I do wonder whether it belongs in a different venue (e.g. a deep learning conference). At the same time, these kinds of empirical works sometimes don't find an easy home in those venues, and I think the neuroscience appeal of the current work is probably enough to justify putting it in this journal.

A few comments / questions (in no particular order):

Hybrid Images

• The use of hybrid images should probably come with a citation to the original paper where these were introduced: Oliva, A., Torralba, A., & Schyns, P. G. (2006). Hybrid images. *ACM Transactions on Graphics (TOG)*, *25*(3), 527-532.

• The hybrid images / reversal paradigm is an elegant way to probe whether a model is leveraging high or low frequency information, but I would imagine that in many cases, models will simply fail (i.e. classify the image as some category other than the one represented in the high or low channel). My understanding is that the authors are only looking at the relative probabilities for the two classes of interest, but other classes might have higher probabilities. Is this a rare condition, or relatively common?

• Is there an advantage to constructing hybrid images vs. simply low-/high-/band-passing the images? Does inducing a conflict serve some purpose?

Other approaches to making models more "brain-like"

• While the paper focuses primarily on models that are regularized to be more brain-like, there are other neuroscience-inspired approaches that at least warrant some discussion, such as adding neuron-like stochasticity to responses (e.g. Dapello et al. NeurIPS 2021 "Neural Population Geometry Reveals the Role of Stochasticity in Robust Perception").

Preprocessing-based (blur, PCA) models

• The authors introduce simple blurred and PCA preprocessing modules to show that a degree of robustness can be achieved by simply removing high frequency information. While these models are introduced halfway through the paper in the context of the reversal/hybrid images analyses, I was curious to what extent these models were robust to corruption and adversarial perturbation (e.g. the analyses presented in figure 2). I'm further curious whether some of the advantage of regularizing with mouse neural data is basically just effectively imposing low pass filtering inside the model (mouse visual acuity is extremely poor, after all). Can the robustness advantages of the mouse-regularized model be explained through blurring? Is there more to this regularization, or could you achieve the same result with blurring? I thought it was also interesting that in Figure 2f, the power spectrum of monkey-regularized adversarial attacks did not show a clear low-frequency preference.

Frequency preference of models vs. frequency vulnerability of attacks

• In a few cases, I found the language of the paper to play somewhat fast and loose with the question of whether a *model* preferred a particular frequency band, or whether attacks against the model preferred different frequency bands (e.g. page 3: "We observed that the mouse and monkey neurally regularized models contain relatively higher low-frequency components than the baseline model"… this is referring to the spectrum of the attack, not the models per se). There's a little bit of subtlety here, insofar as a models robustness against attacks in a particular frequency band can be independent of what information it primarily uses to make decisions. While removing information in a frequency band will certainly limit the utility of attacks in that frequency band, the vulnerability to an attack in a particular frequency band can also depend on the representational geometry in that band. We know, for instance, that humans can take advantage of high frequency information, while not apparently being vulnerable to corruption in these bands.

Discussion topics

At a high level, I wasn't fully clear whether the authors are claiming that *today's* more brain-like models achieve robustness through preference for low frequencies (which might be perceived as a cheat of sorts), or whether preference for lower frequencies is in some sense the "right" thing to do. Is it a sign of how far we still are from understanding that our robust models focus on low frequency information, or is that actually a desirable thing for a model to do?

At a deeper level, it is also the case that models needn't process high and low frequency information independently and could integrate it in a nonlinear way (for instance, a model could leverage high frequency information when it is consistent with low frequency information, but ignore it when it is not). I'd love to see a little more nuance in the discussion around topics like this.

Minor Comments

• pg. 1: "Recent work has shown that machine learning models which are encouraged" -> "that are encouraged"

• pg. 2: Safari -> Safarani

• Figure 5a: the red and pink lines are almost impossible to tell apart

• Page 7, top: “All of this seem to indicate that robustness to different datasets might be achieved trough different methods but low-frequency preference seem to be a key component of current robust models. “ -> “through”… someone should run a spell checker on this whole manuscript…
