# Supplementary material for: Robust deep learning object recognition models rely on low frequency information in natural images
Source: PLoS Comput Biol. 2023 Mar 27;19(3):e1010932. doi: 10.1371/journal.pcbi.1010932 (PMC10079058; doi:10.1371/journal.pcbi.1010932)
Supplement: Attachment — Submitted filename: rebuttal.pdf [file pcbi.1010932.s004.pdf]

Revision notes for "Robust deep learning object recognition models rely on low frequency information in natural images"

Anonymous Authors

ABSTRACT

We made major revisions to address questions and concerns from all three reviewers, and conducted new experiments to add new results and analysis. Responses to the major comments are listed in this document, with revisions highlighted in the marked version of new submission.

We thank the reviewers for their thoughtful comments and feedback. The reviewers think our main conclusion is "supported by the presented evidence", and consider our results "valuable for understanding of robustness" and "a useful contribution to the literature". We address some shared concerns by discussing further about the differences between the mouse regularized models and the monkey regularized one, the biological evidences supporting the association between brain-likeness and frequency bias, the use of hybrid images in our experiments, the relationship between our analysis and other approaches of making models brain-like. We additionally trained a series of 'blur' ResNet-50 models on ImageNet dataset, analyzed their robustness and frequency preference. We show the results are consistent with our observation from CIFAR10 dataset. We also performed several new analysis proposed by the reviewers and included them in the appendix.

R1: Low frequency preference of mouse regularized model is stronger than that of monkey regularized model, what is the key factor of making two neural models so different? Why is the effect weak in monkey regularized one?

We agree with the reviewer that there are many different factors in obtaining the mouse regularized model and the monkey regularized one, and to clearly identify the impact of each individual factor it is best to conduct systemic comparison among all hyper-parameters. However it is a bit out of our scope to perform hyper-parameter comparison to address this question completely, and we instead focus on analyzing the properties of two published models.

That being said, we do speculate the key difference between mouse- and monkey-regularized model is mainly due to their representation itself. Specifically, monkey V1 representation perhaps encodes some salient and robust visual features but not low-spatial frequency. Detailed analysis and discussions can be found in the original monkey regularization paper Safarani et al. (2021). While analysis on mouse V1 representation suggests a clear dominance of low-frequency information (Fig. 10 and 11 in Appendix). We added some discussion in this revision.

R1: Apply blurring preprocessing on models and dataset other than ResNet-18 and CIFAR10, *e.g.* ResNet-50 on ImageNet.

At first we did not successfully train ResNet-50 models with blurring preprocessing on ImageNet, because we attempted to train 'blur' models from scratch without using any pre-trained models, but it took weeks to adjust training hyper-parameters and the model performance was never reasonably good. During the revision we changed our strategy by initializing 'blur' model parameters with a pre-trained baseline ResNet-50 model, *i.e.* copy all layers except the first blurring layer, and fine-tuning the models to accommodate the additional preprocessing layer. Models trained in such way do reach reasonable performance (71% top-1 accuracy) since they are jump-started with a good backbone. However we believe it also induces a bias towards the baseline model which has its own frequency tuning properties. We discussed about this potential pitfall in the result section.

We included these 'blur' ResNet-50 models in the revision (Fig. 6), and compared the one with 'Goldilocks' blurring parameter σ with other models on ImageNet. Performing extensive adversarial attacks on such models is very time-consuming so we only analyzed the adversarial robustness for the selected model (green dot in Fig. 6a).

R1: How about the variance of frequency preference measures due to different random seeds during the network training?

We trained 5 new models with the same architecture, ResNet-18 model with a fixed blurring layer, on the CIFAR10 dataset, and computed the half power frequency $f_{0.5}$ and reverse frequency f_{rev} for each. We found that the variance due to random seeds of both metrics are small, compared with the differences between different architectures. The new results are added in the appendix with more details and discussions.

R1: Caption of Fig. 4b and 5a mentioned dotted lines.

For adversarially robust models and common corruption robust models in Fig. 4b and 5a, each individual model was plotted by dotted thin lines originally, which was not visually obvious. Due to the similarity among individual models, some dotted

lines overlap with others greatly and are occluded by the thick solid line that represents the group average. We now changed the dotted lines to solid lines with light colors, and revised the caption accordingly.

R1: Can the author discuss whether mouse visual system features can explain the low frequency preference in mouse regularized model?

Previous physiological experiments reported that a typical mouse V1 neuron's preferred spatial frequency is around 0.04 cycles per visual degree. In the mouse regularization work, the image shown to the animal is about 67.5 degrees, which means the V1 preferred spatial frequency is 2.7 cycles per image, which we consider as low frequency since each image usually contains one single object. We added the computation and comparison with monkey data in the discussion section as well.

We also added a paragraph in the discussion section to further address the analysis on neural manifold included in the appendix. We decomposed the mouse V1 neural manifold into orthogonal dimensions, and analyzed the spatial tuning property along each major component. We argue that the low frequency preference was present in the neural manifold and got inherited by mouse regularized models.

R1: If the low frequency preference cannot fully explain the robustness in mouse regularized model, can the author explain other possible reasons?

Broadly speaking, we think the robust models pick up robust features of the images and make decisions based on them. However what exactly is a robust feature is unanswered, by no means it has to be defined by spatial frequency purely. Features that are invariant over different perturbations can in principle be of high spatial frequency, but characterized by some higher-order statistics (Karklin and Lewicki, 2009).

R1: Comparison with the paper "Increasing neural network robustness improves match to macaque V1 eigenspectrum, spatial frequency preference and predictivity".

We thank the reviewer for referring to this recent research, which also addresses the relationship between brain-likeness and robustness, but from another perspective. We added some discussions in the revision.

R2: How often does the model predict hybrid images as neither the category of low-frequency component nor that of high-frequency component?

When the hybrid image is ambiguous, the predicted category by the model can indeed be inconsistent with either component. We looked at the baseline ResNet-18 model in Fig. 3b at the mixing frequency $f_{\text{mix}} = 0.55$, where probability of reporting low- and high-frequency component category are roughly the same. With $p_{\text{low}} \approx p_{\text{high}} \approx 25\%$ in this case, about 50% of the total images are predicted as a third category. We also observed that the model confidence, *i.e.* the probability of predicted category is lowest at such ambiguity level. We added a new figure and some analysis in the appendix.

R2: Is there an advantage of using hybrid images over using low-/high-passing images? Does introducing a conflict serve some purpose?

We use hybrid images instead of low-/high-passing images to keep the artificial images resemble as much as possible to the natural images in terms of image statistics. For example, low-/high-passing images were used in Yin et al. (2019), and is is essential to rescale the constructed images to proper mean pixel values and standard deviation. Even in that case, the pixel value histogram of the probing images still differ a lot from natural images. We believe such images deviate too far from the data distribution that models are trained on. Hybrid images on the other hand have the same pixel value distribution as natural images, which guarantee at least the first order statistics to be calibrated. At the same time, since hybrid images are constructed by stitching Fourier components from different seed images, its power spectrum is still close to natural images. Based on the above reasons, we feel hybrid images are better suited to probe the trained models than just low-/high-passed components.

In addition, since our major metric of model evaluation is their classification accuracy under different image perturbation, we introduce category conflict to focus on the robust features associated with classifications specifically.

R2: How about other approaches to make the model brain-like such as adding stochasticity?

We added a paragraph in the discussion section to talk about the relationship between our work and Dapello et al. (2021). In short, stochasticity generally does not change the model representation, and the induced robustness are usually related to gradient masking which requires different evaluation protocols compared to evaluating deterministic models (Athalye et al. 2018).

R2: Are mouse regularization simply filtering out high-frequency information? Low frequency bias in monkey regularized model is small.

We do not claim the robustness by mouse regularization is simply due to filtering different Fourier components, though such linear mechanism may lead to approximately same effect. Our analysis on mouse V1 manifold suggests there are more structures to the neural features than merely spatial frequency. We also added discussions about the monkey regularized models, please see our response to reviewer 1's questions for more details.

R2: Differences between frequency preference of model and the frequency vulnerability of attacks.

There is indeed subtle differences of these two concepts, however we believe they are practically the same. The preferred

frequency or feature of a model determines which direction in the image space the model is most sensitive to, namely which direction of perturbation is most effective in changing the model's outputs. Naturally, the minimal adversarial attacks are optimized to align with those features.

R2: Colors in Fig. 5a is difficult to tell apart.

We changed the color of PCA models to yellow in the revision.

R3: Introduce blur models earlier in the paper to better demonstrate the computational hypothesis.

We thank the reviewer for the suggestion, and adjusted order of our texts slightly. Since we are not proposing low-pass blurring as the mechanism of the brain encoding robust features, we want to emphasize on the descriptive aspect of our understanding on neural features. Gaussian blurring is one simple mechanism for introducing the association between low frequency preference and robustness we observed in all models, but that is not the only way of achieving higher robustness.

R3: Why evaluate model robustness against common corruptions by averaging over different types and severity levels? Can they be analyzed separately?

We use the average accuracy over all corruption types and severity levels as the evaluation of model robustness for purely practical reasons. Since we are comparing different models, it will be easier to use one number to describe one model. It is common practice to report model accuracy on the full CIFAR10-C or ImageNet-C dataset, which contains a fixed number of different image corruptions.

We agree with the reviewer that more insightful comparison should be made on each individual corruption type and level. We regrouped the classification accuracy of models trained on CIFAR10, and plotted them against model frequency preference separately. The new results are included in the appendix. We found that though the exact dependency of robustness on frequency preference f_{rev} differ for different corruptions, the overall alignment towards 'blur' models always hold approximately.

R3: What do we know about whether the biological circuits indeed blur the images?

We added discussions on the mouse and monkey V1 neuron tuning properties measured by physiological experiments, please see our response to reviewer 1's questions for more details.

R3: Please include more details about the overall procedure.

We added a new section in the appendix with more implementation details, and also released all codes via GitHub. The two specific questions raised by the reviewer is also answered below.

The "full testing dataset" means the testing set of grayscale CIFAR10 (mouse regularized model and baseline) and the testing set of grayscale TinyImageNet (mouse regularized model and baseline) respectively.

Comprehensive evaluation of adversarial robustness is time consuming, so we only performed targeted attacks on a fixed subset of testing set images. The attack target class for each selected image is also randomly decided and fixed, so that we can compare different models in a fair manner.

R3: How did the green curve in Figure 5b come about?

The green curve was a second order polynomial fit on 'blur' models with different blurring parameters. We now plot all individual models explicitly with 'x' markers, and updated the caption of Fig. 5. Each 'blur' model for CIFAR10 is independently trained from scratch.

R3: Is it better to show accuracy decrease instead of absolute accuracy in Fig. 5 and 6? The term "corruption accuracy" is a misnomer and slightly confusing.

The reason we use raw accuracy instead of the accuracy drop is we think the latter is more misleading. An extreme case would be a chance-level classifier, whose accuracy does not decrease on corrupted images. It gives an illusion of perfect robustness, though the model is not useful in any way. We feel it is better to present model accuracy on corrupted images directly, and report the clean performance in Table 2 and 3 for references.

The y-axis label is changed to 'CIFAR10-C acc. (%)' and 'ImageNet-C acc. (%)' respectively, and the captions are revised accordingly.

R3: Color bars missing in Figure 2 and 4.

A color bar with label is added to Fig. 2 and 4. Since each inset figure is normalized separately, we only used uneven ticks to indicate that the heat maps are colored by logarithm scale but did not add tick labels. Captions are revised accordingly.
