# Supplementary material for: Robust deep learning object recognition models rely on low frequency information in natural images
Source: PLoS Comput Biol. 2023 Mar 27;19(3):e1010932. doi: 10.1371/journal.pcbi.1010932 (PMC10079058; doi:10.1371/journal.pcbi.1010932)
Supplement: Attachment — Submitted filename: rebuttal.pdf [file pcbi.1010932.s005.pdf]

Revision notes for “Robust deep learning object recognition models rely on low frequency information in natural images”

Zhe Li[1,*,‡], Josue Ortega Caro[1,*], Evgenia Rusak[2], Wieland Brendel[2], Matthias Bethge[2], Fabio Anselmi[1], Ankit B. Patel[1,3,4,+], Andreas S. Tolias[1,3,4,+,‡], and Xaq Pitkow[1,3,4,+,‡]

[1]Department of Neuroscience, Baylor College of Medicine, Houston, 77030, USA
[2]University of Tübingen, Germany
[3]Department of Electrical and Computer Engineering, Rice University, Houston, 77005, USA
[4]Center for Neuroscience and Artificial Intelligence, Baylor College of Medicine, Houston, 77030, USA
[*]co-first authors
[+]co-senior authors
[‡]co-corresponding authors

ABSTRACT

We conducted new experiments to include more baseline models of various architectures, updated figures with the new robustness analysis results. Responses to the latest comments are listed in this document, with revisions highlighted in the marked version of new submission.

We thank the reviewers for their thoughtful comments and feedback in the second round. The only main concern was about the lack of diversity of baseline models, therefore we added robustness analysis to five new baseline models for CIFAR10 dataset with various architectures. Details of these models are included in Appendix Table 2. Figure 4 and 5 in the main text along with figure 14 and 15 in the appendix are updated accordingly.

The latest results show that though the baseline models we newly included differ greatly in their architectures (‘ResNet’, ‘WideResNet’, ‘VGG’, ‘MobileNetV2’, ‘ShuffleNetV2’, ‘RepVGG’), their robustness against common corruptions and adversarial attacks are similar. In addition, the spatial frequency preferences from our analysis are also similar, supporting our main conclusion that model robustness is largely explained by its frequency preference at least in CIFAR10 models.

Responses to other minor comments are listed below:

R1: The reason why models are robust is different for those trained on low or high resolution images.

We thank the reviewer for the comments and we agree that the simple view of spatial frequency does not explain every aspect of robustness in models trained on large images. There might be high-frequency but still reliable visual features that robust models (as well as biological visual system) are using. More discussions are included in the second and third paragraph of the discussion session. We would like to delve into this problem in future research.

R2: The second part of the paper should report means and standard deviations like the first part.

We presented the comparison of all public models via figures, and felt it was visually more clear by omitting error bars of each marker in the scatter plot.

In this revision, we calculated standard error of means for the minimal adversarial attack size ε and added them to the appendix. We also computed the standard deviation reversal frequency f_{rev} using different hybrid image datasets as in ‘Hybrid image experiment’ session. The results range from 3e-4 to 2e-3, all negligible with respect to the inter-model difference (Fig. 5 and 6). Model accuracy on the corruption datasets and the half power frequency $f_{0.5}$ are both defined over a fixed set of images, therefore have no error caused by randomization.

R2: Are there multiple mouse models or just one? Why not just one VGG19 mouse model? It would be helpful for the reader to very briefly clarify in the manuscript what mouse/monkey regularized models are.

We apologize for the confusion about number of models. There were in fact multiple mouse regularized models used in the original study (Li et al. 2019), using different random initializations. However we only used one for analysis in this study, *i.e.* the one in Fig. 2 and 3. We have modified the plural terms in the text to avoid confusion.

VGG19 model was used in a separate study (Safarani et al. 2021) for monkey response regularization. We do not have a VGG19 model trained with mouse data.

The methods to train a mouse regularized model and a monkey regularzied one were described in the second paragraph of 'Neural regularization boosts model robustness' session.

R2: 'adv' and 'crp' are not explained in the figure legends.

We modified the legend of figure 4 and 5 to explain these abbreviations.
